# MicroRNA-320 suppresses colorectal cancer by targeting SOX4, FOXM1, and FOXQ1

**DOI:** 10.18632/oncotarget.8937

**Published:** 2016-04-22

**Authors:** Radhakrishnan Vishnubalaji, Rimi Hamam, Yue Shijun, Omar Al-Obeed, Moustapha Kassem, Fei-Fei Liu, Abdullah Aldahmash, Nehad M. Alajez

**Affiliations:** ^1^ Stem Cell Unit, Department of Anatomy, College of Medicine, King Saud University, Riyadh, Kingdom of Saudi Arabia; ^2^ Princess Margaret Cancer Centre, University Health Network, Toronto, ON, Canada; ^3^ Colorectal Research Center, Department of Surgery, King Khalid University Hospital, College of Medicine, King Saud University, Riyadh, Saudi Arabia; ^4^ KMEB, Department of Endocrinology, University of Southern Denmark, Odense, Denmark; ^5^ Danish Stem Cell Center (DanStem), Panum Institute, University of Copenhagen, Copenhagen, Denmark; ^6^ Prince Naif Health Research Center, King Saud University, Riyadh, Kingdom of Saudi Arabia

**Keywords:** colorectal cancer, miR-320, SOX4, FOXM1, FOXQ1

## Abstract

Colorectal cancer (CRC) is the third most common cancer causing high mortality rates world-wide. Delineating the molecular mechanisms leading to CRC development and progression, including the role of microRNAs (miRNAs), are currently being unravelled at a rapid rate. Here, we report frequent downregulation of the microRNA miR-320 family in primary CRC tissues and cell lines. Lentiviral-mediated re-expression of miR-320c (representative member of the miR-320 family) inhibited HCT116 CRC growth and migration *in vitro,* sensitized CRC cells to 5-Fluorouracil (5-FU), and inhibited tumor formation in SCID mice. Global gene expression analysis in CRC cells over-expressing miR-320c, combined with *in silico* prediction identified 84 clinically-relevant potential gene targets for miR-320 in CRC. Using a series of biochemical assays and functional validation, SOX4, FOXM1, and FOXQ1 were validated as novel gene targets for the miR-320 family. Inverse correlation between the expression of miR-320 members with SOX4, FOXM1, and FOXQ1 was observed in primary CRC patients' specimens, suggesting that these genes are likely *bona fide* targets for the miR-320 family. Interestingly, interrogation of the expression levels of this gene panel (SOX4, FOXM1, and FOXQ1) in The Cancer Genome Atlas (TCGA) colorectal cancer data set (319 patients) revealed significantly poor disease-free survival in patients with elevated expression of this gene panel (*P*-Value: 0.0058). Collectively, our data revealed a novel role for the miR-320/SOX4/FOXM1/FOXQ1 axes in promoting CRC development and progression and suggest targeting those networks as potential therapeutic strategy for CRC.

## INTRODUCTION

Colorectal cancer (CRC) is the third most common type of cancer in incidence, as well as the fourth most common cause of death for both genders around the globe [1]. Conventional chemotherapeutic strategies for CRC involves the use of highly toxic drugs with many undesirable side-effects [2, 3], underscoring the need to identify novel biomarkers or combinational therapies for early diagnosis, as well as improved disease stratification and treatment choices. Recently, it was shown that gene expression is orchestrated at the posttranscriptional level by small noncoding RNA species. One prominent class of noncoding RNAs are microRNAs (miRNAs) that have been intensely investigated in various biological systems [4]. Inter-regulation between messenger RNA (mRNA) and miRNA is a complex cellular process; insights into these interactions facilitate the understanding of the fine-tuning of transcriptional and translational outputs, and developmental processes [5]. MiRNAs have been implicated in a myriad of fundamental cellular and physiological processes including cellular differentiation, proliferation, survival, motility, apoptosis and stem cell maintenance [6–8]. Using mRNA-miRNA transcriptomics data in conjunction with *in silico* target prediction algorithms, plus functional validation studies is a potent strategy for the identification of novel mRNA-miRNA regulatory networks in different human diseases [9–11].

Over the past decade, aberrant expression of different miRNAs (oncomiRs and tumor suppressor miRNAs) have been implicated in driving colorectal cancer progression [8, 10, 12–14]. In particular, our recent data have revealed over 700 potential miRNA-mRNA regulatory networks in colorectal cancer [10]. Notably, the expression level of miR-320 family (miR-320a, -b, -c, -d and -e) were significantly down-regulated in CRC samples compared to adjacent normal mucosa [10].

While the miR-320 family has been described to be involved in several different human malignancies [15–19], to date however; the role of the miR-320 family in CRC has not been fully elucidated. Herein, we took an unbiased approach and identified the biologically and clinically-relevant gene targets for miR-320 family in CRC. Lentiviral-mediated re-expression of miR-320c (representative member of the miR-320 family) inhibited CRC growth *in vitro* and *in vivo*, and sensitized CRC cells to 5-Fluorouracil (5-FU). Global gene expression analysis, *in silico* prediction, and functional validation revealed SOX4, FOXM1, and FOXQ1 as novel gene targets for miR-320 family. We observed an inverse correlation between the expression of miR-320 members with SOX4, FOXM1, and FOXQ1 in CRC patients' specimens, strongly indicating that those genes are *bona fide* targets for miR-320 family.

## RESULTS

### MiR-320 family is downregulated in CRC and their overexpression reduces HCT116 cell growth and migration

**Figure 1:**
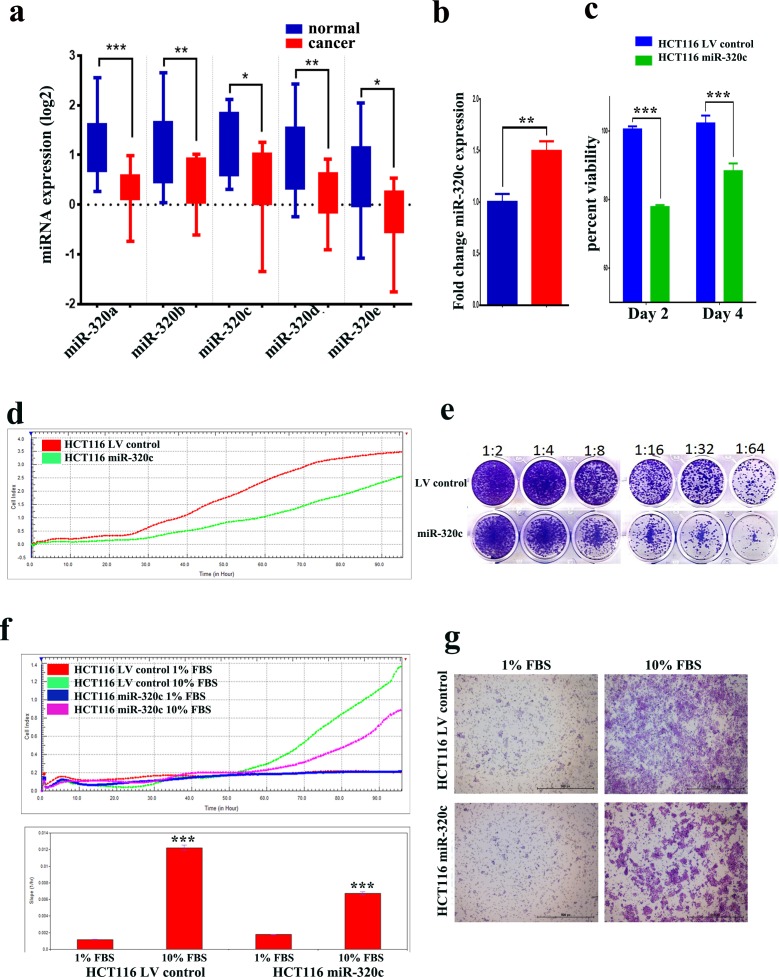
miR-320 family is downregulated in CRC and it suppresses CRC cell proliferation, migration and clonogenicity **a.** Expression of miR-320a, -b, -c, -d, and e in CRC (Log2) compared to adjacent normal tissue based on microarray data. Data are presented as mean ± S.E., *n* = 13. **b.** qRT-PCR quantification of hsa-miR-320c expression in miR-320c HCT116 compared to LV control cells. Data are representative of three experiment and are presented as mean ± S.D., *n* = 3. **c.** Lentiviral-mediated re-expression of miR-320c in HCT116 cells reduces their cell viability. **d.** Real time proliferation assay revealed significant decrease in the proliferation of miR-320c HCT116 compared to LV control cells in a time-dependent manner. **e.** Clonogenic assay showing remarkable reduction in the colony forming capability of miR-320c HCT116 cells compared to LV control cells. Plates were stained with Diff-Quik stain set on day 10. Wells are representative of two independent experiments for each condition. **f.** and **g.** Real time and conventional migration assay showing significant inhibition of cell migration in the miR-320c HCT116 compared to LV control cells. The two-tailed t-test was used to compare different treatment groups. ***p < 0.0005.

Our previous miRNA expression profiling in CRC compared to adjacent normal tissues revealed multiple dysregulated miRNAs, including downregulation of the miR-320 family (miR-320a, -b, -c, -d, and -e) (Figure 1a) [10]. MiR-320c was subsequently used to represent the miR-320 family in the subsequent functional studies conducted using the HCT116 CRC model, which have low levels of miR-320 expression (Supplementary Figure 1). Lentiviral-mediated stable expression of miR-320c reduced the viability of HCT116 colon cancer cells *in vitro* (Figure 1b and 1c). Similar results were also observed when hsa-miR-320c was over-expressed in the SW620 and HCT8 CRC cell lines (Supplementary Figure 2). Similar inhibitory effects were observed when hsa-miR-320a was over-expressed in the SW620 and HCT8 CRC cell lines (Supplementary Figure 3). Real-time proliferation assay revealed a significant reduction in the growth of miR-320c-HCT116 cells compared to LV control cells during 100-hour observation period (Figure 1d). Concordantly, the clonogenic assay also revealed lower number of colonies in the miR-320c-HCT116 compared to LV control cells (Figure 1e), suggesting a strong inhibitory effect of miR-320c on colony formation in the HCT116 model. Similar inhibitory effects were observed on cell migration toward media containing 10% FBS in the miR-320c HCT116 compared to LV control cells employing two independent assays: microelectroic sensor plate assay (Figure 1f) and transwell assay (Figure 1g), implicating a role for this miRNA in migration as well as in proliferation.

### Multiple dysregulated pathways in miR-320c HCT116 cells

**Figure 2:**
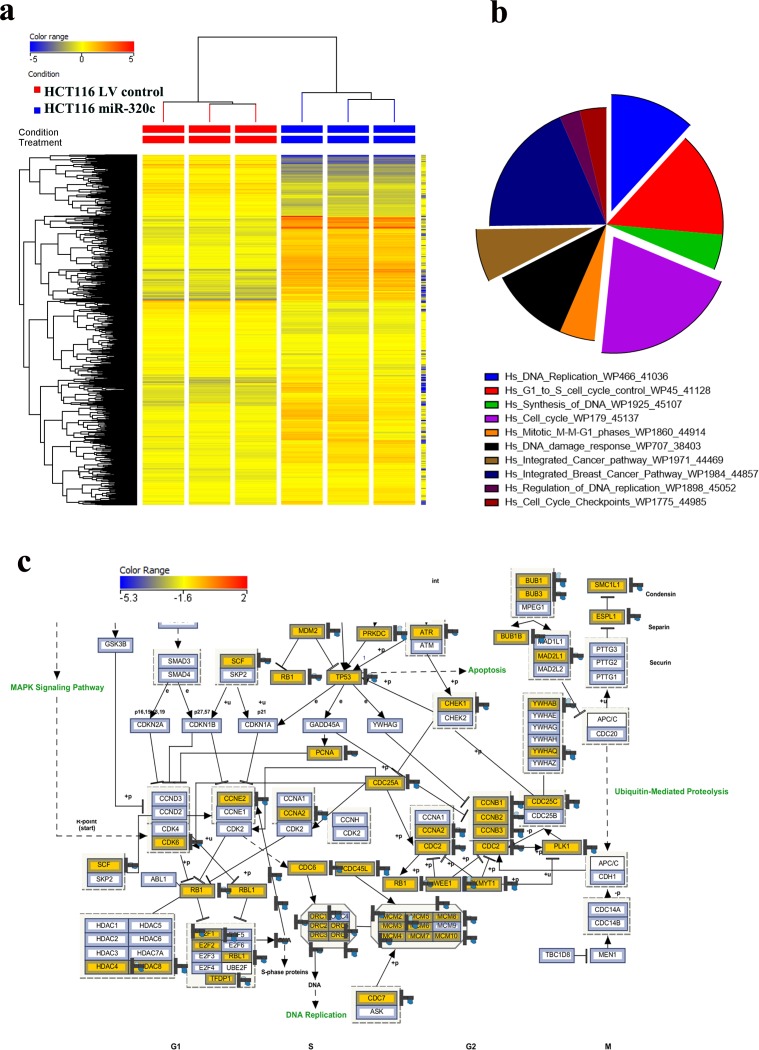
miR-320c regulated multiple cellular processes in HCT116 cells **a.** Hierarchical clustering of miR-320c HCT116 *vs* LV control HCT116 cells based on differentially expressed mRNA levels. Each column represents one replica and each row represents a transcript. Expression level of each gene in a single sample is depicted according to the colour scale. **b.** Pie chart illustrating the distribution of the top 10 pathway designations for the downregulated genes in miR-320c HCT116 cells. The pie size corresponds to the number of matched entities. **c.** The integrated cell cycle pathway is illustrated in panel c.

To unravel the molecular and cellular processes regulated by miR-320c, we performed global mRNA expression profiling comparing miR-320c HCT116 with LV Control cells. As shown in Figure 2a, hierarchical clustering based on differentially-expressed mRNAs revealed clear separation of the two groups. Using significance analysis, we observed 3844 downregulated transcripts in miR-320c HCT116 cells (Supplementary Table 1). The distribution of the top 10 enriched pathway designations for the downregulated genes in miR-320c HCT116 cells are shown in Figure 2b, indicating a role for miR-320c in regulating cell cycle, DNA replication, DNA damage response, and integrated cancer pathways. Illustration of the cell cycle pathway is presented in panel Figure 2c, with matched entities highlighted.

**Figure 3:**
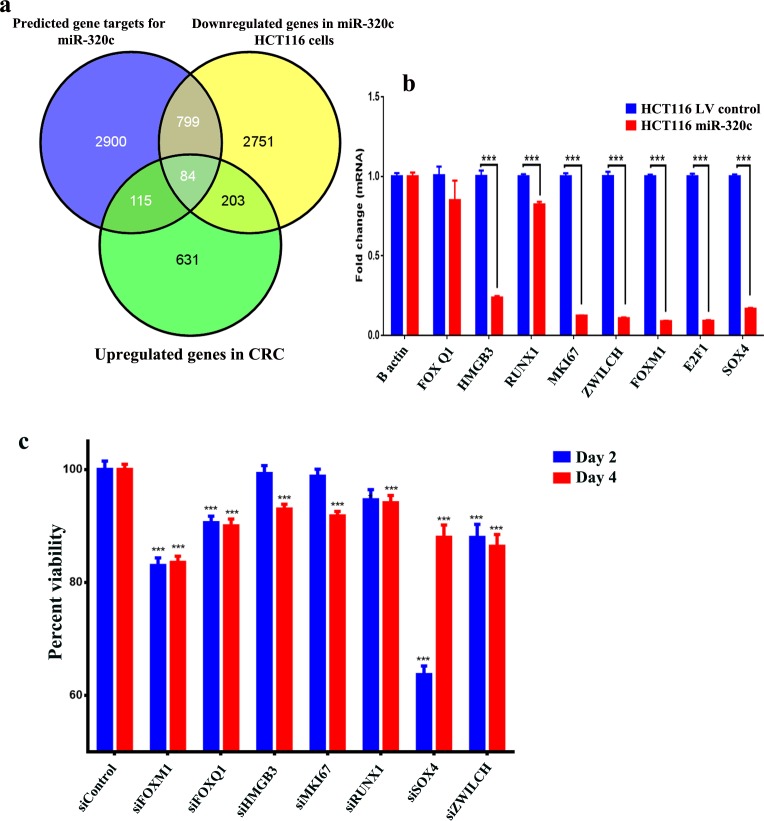
Identification of the clinically-relevant miR-320c target genes in CRC **a.** Venn diagram depicting the overlap between the predicted gene targets for miR-320c (based on TargetScan algorithm) *vs* the downregulated genes in miR-320c HCT116 cells and the differentially upregulated genes in CRC identified from microarray gene expression. **b.** The expression levels of selected genes that were common to all gene lists in (a) were validated using qRT-PCR in miR-320c HCT116 cells. Data are presented as mean ± S.E., *n* = 6. **c.** siRNA-mediated knockdown of FOXM1, FOXQ1, HMGB3, MKI67, RUNX1, SOX4 and ZWILCH led to significant reduction in HCT116 cell viability. Data are presented as mean ± S.E., *n* = 12. ***p < 0.0005. The two-tailed *t*-test was used to compare different treatment groups.

In order to identify the biologically and clinically relevant gene targets for miR-320 family in CRC, we focused on the list of genes that were downregulated in miR-320c HCT116 cells, that were predicted to be targeted by miR-320 family using *in silico* prediction (TargetScan), and those that were also upregulated in primary CRC specimens based on microarray gene expression profiling. Using this strategy, we identified 84 genes as potential miR-320 targets in CRC (Figure 3a, Supplementary Table 2). The expression levels of a selected number of these genes (FOXQ1, FOXM1, HMGB3, RUNX1, MKI67, ZWILCH, E2F1 and SOX4) was subsequently validated using qRT-PCR (Figure 3b). siRNA-mediated knockdown of several of those genes (FOXM1, FOXQ1, HMGB3, MKI67, RUNX1, SOX4, and ZWILCH) led to a significant reduction in HCT116 cell viability, similar to those observed when miR-320c was over-expressed in HCT116 cells, strongly indicating these genes are potential targets for miR-320 family in CRC (Figure 3c).

### miR-320c directly targets SOX4, FOXM1 and FOXQ1 in colorectal cancer

**Figure 4:**
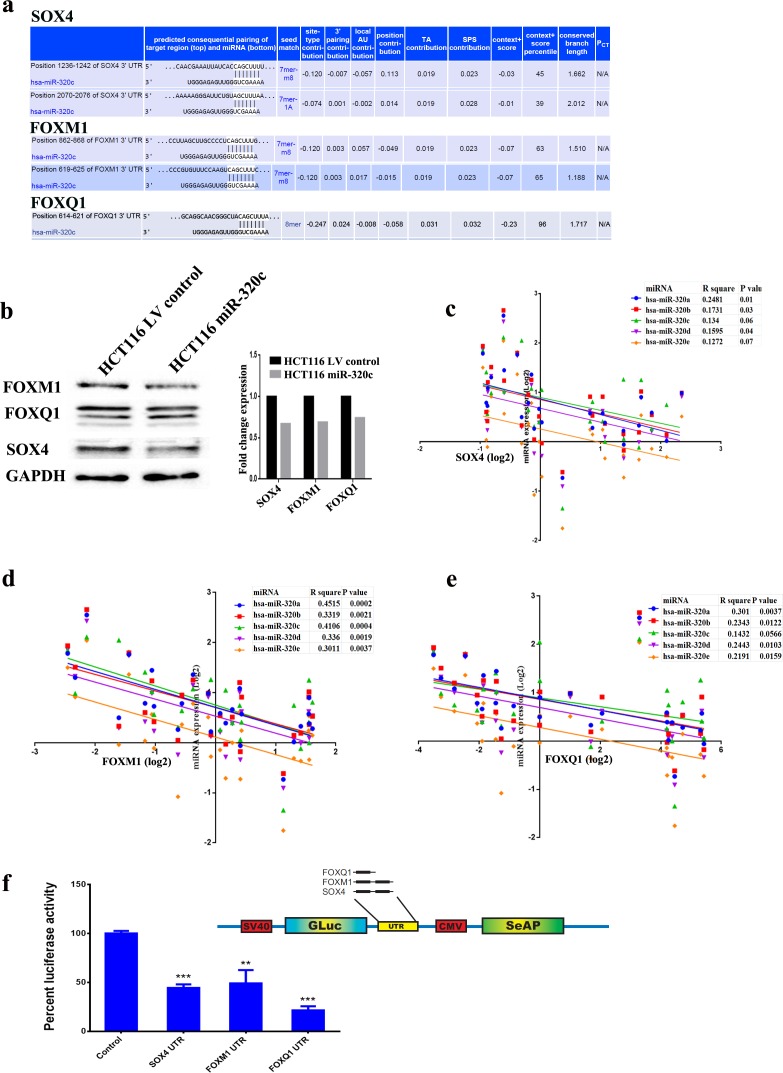
Identification of SOX4, FOXM1 and FOXQ1 as *bona fide* targets for miR-320c **a.** Illustration of the alignment of miR-320c and SOX4, FOXM1 and FOXQ1 3` UTR using TargetScan algorithm indicating potential binding sites. **b.** Immunoblotting showing downregulation of SOX4, FOXM1 and FOXQ1 protein in miR-320c HCT116 compared to control cells. Right panel presents quantification of band intensity. GAPDH was used as loading control. **c.**, **d.** and **e.** Pearson correlation between the expression of target mRNAs (SOX4, FOXM1 and FOXQ1) and different members of the miR-320 family in thirteen pairs of CRC, corroborating SOX4, FOXM1 and FOXQ1 being a *bona fide* targets for miR-320 family in CRC. **f.** Luciferase-based reporter assay illustrating the direct interaction between miR-320c and the 3`UTR from SOX4, FOXM1 and FOXQ1. Data are presented as mean ± S.E., *n* = 5 from two experiments. The two-tailed t-test was used to compare different treatment groups.

Three genes (SOX4, FOXM1, and FOXQ1) were subsequently chosen for further investigation based on their known role in colorectal or other human cancer types. Alignment of miR-320c and gene targets 3` UTR using TargetScan algorithm indicated two potential binding sites for SOX4 (position 1236-1242 and 2070-2076), two binding sites for FOXM1 (position 862-868 and 619-625), and one binding site for FOXQ1 (position 614-621) (Figure 4a). Correspondingly, SOX4, FOXM1 and FOXQ1 protein levels were decreased in miR-320c HCT116 compare to the LV control cells, indicating suppression of those genes at the protein level (Figure 4b). Importantly, the expression of SOX4, FOXM1, FOXQ1 was inversely correlated to miR-320 levels in primary paired CRC patient samples, strongly indicating that all three genes are likely to be *bona fide* targets for miR-320 (Figure 4c, 4d and 4e). Direct interaction between miR-320c and the 3`UTR from SOX4, FOXM1 and FOXQ1 was subsequently validated using a luciferase-based assay (Figure 4f). Taken together, these results demonstrated that downregulation of miR-320 family is a potential mechanism leading to the upregulation of SOX4, FOXM1 and FOXQ1 in CRC.

### Forced expression of miR-320c increase sensitivity of CRC cells to 5-fluorouracil

**Figure 5:**
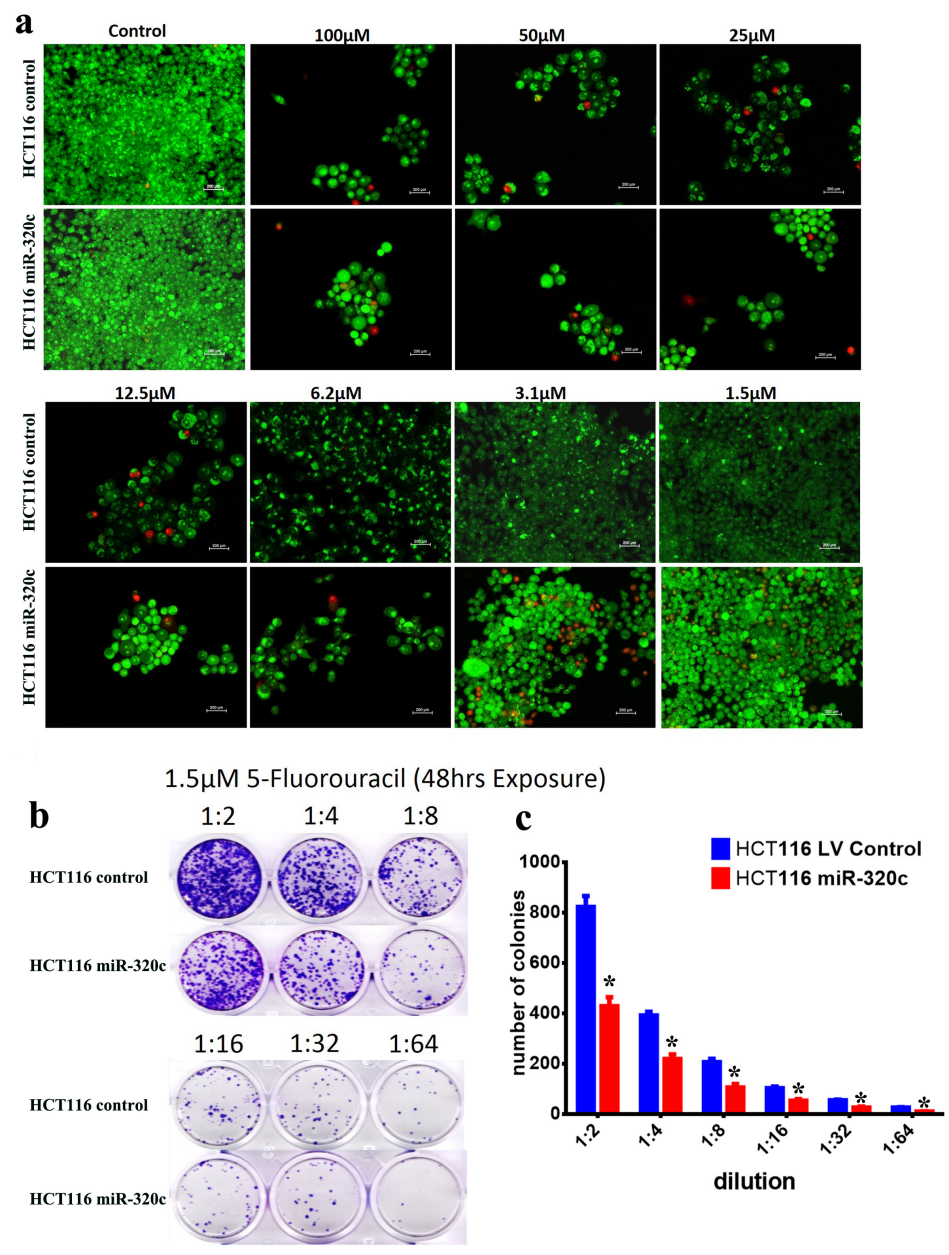
miR-320c sensitize CRC cells to 5-fluorouracil **a.** Representative fluorescence images of miR-320c and LV control HCT116 cells (±different concentration (1.5-100 μM) 5-fluorouracil). Cells were stained with acridine orange/ethidium bromide to detect apoptotic (cells with green condensed chromatin) and necrotic cells (red) **b.** Representative clonogenic assay showing reduced clonogenicity of LV-miR-320c compared to LV control HCT116 cells (± 1.5 μM 5-fluorouracil). Plates were stained with Diff-Quik stain set on day 10. **c.** Cells were treated as in (b) in 6-well plate then the number of colonies was counted on day 10. Data are presented as mean colony number ± S.D., *n* = 2. *p < 0.05.

Our pathway analysis of downregulated genes in miR-320c-HCT116 cells revealed response to DNA damage as the sixth affected pathway, suggesting that over-expression of miR-320c might sensitize cancer cells to DNA damage inducing agents (Figure 2b). We therefore used the Acridine orange/Ethidium bromide assay to measure cell death in miR-320c HCT116 compared to LV control HCT116 cells in the presence of 5-FU. The concentration of 5-FU >3.125 μM was highly toxic to both cell groups; however using a lower concentration (< 3.125μM) induced more cell death in miR-320c HCT116 compared to LV control HCT116 cells on day 5 (Figure 5a). Concordantly, we also observed fewer colonies in miR-320c HCT116 + 5-FU compared to LV control HCT116 + 5-FU cells, suggesting that this combination might well be targeting the colony forming ability of these cells (Figure 5b and 5c).

### miR-320c expression suppressed CRC tumor growth *in vivo*

**Figure 6:**
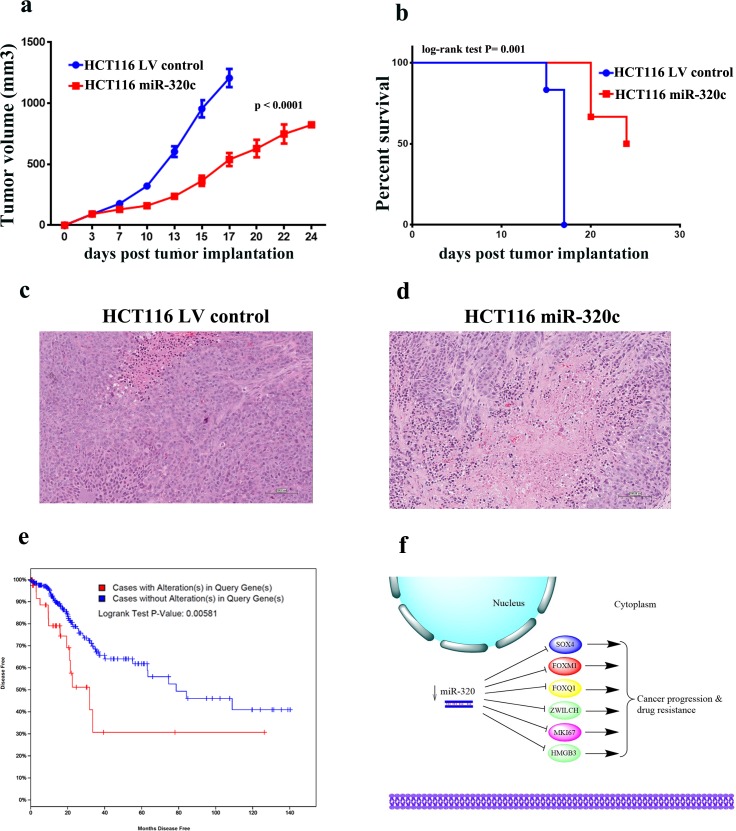
miR-320 expression suppresses CRC growth *in vivo* **a.** Tumour formation in SCID mice after subcutaneous injection of HCT116 cells stably-expressing miR-320c or LV control cells. Data are presented as mean (tumor volume) ± S.E., *n* = 6. Two-way ANONA analysis was used to compare the two growth curves. **b.** Mice in (a) were monitored and surviving fraction was plotted as function of time from tumor implantation. **c.**, **d.** Representative histopathological examination of xenograft tumors from miR-320c and control HCT116 cells. FFPE sections were stained with haematoxylin and eosin stain. (Bar = 100 μm). **e.** Kaplan-Meier curves illustrate the duration of disease-free survival according to the expression of SOX4, FOXM1, or FOXQ1 panel in a cohort of 319 colorectal cancer patients. Using log-rank analysis; expression of SOX4, FOXM1, and FOXQ1 was associated with poor disease-free survival (*p* = 0.005). **f.** Schema illustrating the role of miR-320 family in CRC. Downregulation of miR-320 leads to up regulation of several genes involved in promoting CRC progression and drug resistance.

To demonstrate the biological relevance of miR-320 on CRC tumorigenesis *in vivo*, miR-320c HCT116 or LV control HCT116 cells were implanted into immune-deficient SCID mice that were monitored for tumor formation. Remarkable reduction in tumor growth was observed in the miR-320c HCT116 implants, thereby corroborating the *in vitro* data of miR-320c inhibition of clonogenicity (Figure 6a). Concordantly, mice implanted with the miR-320c HCT116 cells showed better survival compared to mice implanted with control cells (*p* = 0.001, Figure 6b). Likewise, histological examination of xenograft tumors revealed a much greater degree of cell death (necrosis and apoptosis) in the miR-320c HCT116 group (Figure 6c and 6d). Interestingly, interrogation of the expression of this identified gene panel (SOX4, FOXM1, and FOXQ1) in The Cancer Genome Atlas (TCGA) Colorectal cancer data set revealed significantly shorter disease-free survival in patients with elevated expression of these genes (Logrank Test P-Value: 0.00581, Figure 6e).

## DISCUSSION

Carcinogenesis is a multi-step process arbitrated by multifaceted cascades of molecular events governing genomic alterations resulting in the cancer phenotype including uncontrolled cell proliferation [20]. Cumulative evidence revealed a functional involvement of specific miRNAs in cellular alteration and oncogenesis, and documented a pivotal pro- and anti- tumor role for specific miRNAs both *in vitro* and *in vivo* [21, 22]. Also, miRNA expression signatures have been proposed as diagnostic, prognostic or predictors of therapy response in different human cancers [21, 22]. Our recent work on CRC revealed multiple miRNA-mRNA regulatory networks, and have proposed loss and gain of several miRNAs as a key mechanism leading to CRC development and progression [10]. In the current study, we have identified several members of the miR-320 family to be downregulated in CRC. While a number of studies have implicated miR-320 family in different human cancers, none to date has conducted such in-depth investigation of the role of this family of miRNAs in CRC. Therefore, in this current study, we had utilized an integrative approach utilizing stable lentiviral-mediated miRNA expression, gene expression microarrays, *in silico* prediction algorithms, *in vitro* and *in vivo* functional studies to identify clinically-relevant novel gene targets for this family of miRNAs in CRC. Overexpression of miR-320c inhibited colon cancer cell proliferation, migration, colony formation, and *in vivo* tumor formation, elucidating a potent tumor suppressive role for this microRNA in CRC cells. Moreover, our data are the first to reveal FOXQ1, and SOX4, and possibly HMGB3, MKI67, ZWILCH as novel targets for miR-320 family in CRC. Furthermore, our data documented an inverse correlation between miR-320 family (miR-320a, b, c, d and e) with FOXM1, FOXQ1 and SOX4, in 13 pairs of colon cancer tissues, thereby corroborating those genes as likely bona fide targets for miR-320 family and further implicating the miR-320/SOX4/FOXM1/FOXQ1 axes in CRC. Interestingly, the clinical importance of this gene panel was further validated in the TCGA colorectal data set (319 patients) where elevated expression of this gene panel (SOX4, FOXM1, and FOXQ1) correlated with significantly shorter disease-free survival (Figure 6e).

Our current study corroborated previous studies that reported biological effects of miR-320 family in CRC. However, these studies have identified a different set of target genes. Sun et. al. reported that miR-320a inhibits the growth of CRC by targeting b-catenin signalling pathway [13]. Also Rac1 was identified as a direct target for miR-320a in CRC [23]. Interestingly, Zang et. al. reported downregulation of miR-320d in colon cancer stem cells (CD133+) compared to CD133- cells in HT-29 CRC cells, which is concordant with our data implicating miR-320 in regulating CRC tumor initiating cells using *in vitro* clonogenic and *in vivo* tumor formation assays [14].

In addition to CRC, aberrant expression of miR-320 has been reported in several other types of human cancers. For instance, miR-320 was reported downregulated in malignant cholangiocarcinoma, which subsequently was found to negatively regulate Mcl-1 or Bcl-2 (anti-apoptotic molecules) expression; in turn associated with chemotherapeutic drug-triggered apoptosis [24, 25]. In prostate cancer, overexpression of miR-320 blocked the Wnt/b-catenin pathway and reduced the cancer stem cell phenotype in the CD44^high^ tumor-initiating cells [17]. Concordant with our data, Want *et al* recently implicated miR-320 in chemo-sensitivity by targeting FOXM1 *in vitro*, a critical gene which plays a principle role in colon, lung, and breast cancer tumor initiation and progression [26–28]. Similarly, other investigators have also shown that FOXQ1 played a key role in nasopharyngeal carcinoma, targeted by miR-506 and miR-124 [29, 30]; likewise, HMGB3 in breast cancer is targeted by miR-205 [31]; and MKI67 in hepatocellular carcinoma, targeted by miR-519d [32].

In conclusion, based on our newly-generated data, we propose a model (see schema in Figure 6f), whereby loss of miR-320 family leads to increased levels of several miR-320 target genes, resulting in CRC progression, and drug resistance. Therefore, we would propose that miR-320 mimics might serve as a potential therapeutic strategy in the future management of CRC.

## MATERIALS AND METHODS

### Cells lines and tissue culture

The human colorectal cancer (HCT116) cell line was obtained and subsequently was authenticated by Genetica DNA Laboratories, Inc. Burlington, (NC, USA). Cells were maintained in DMEM supplemented with 10% fetal bovine serum (Gibco-Invitrogen, Waltham, MA, USA) and 100 mg/L penicillin/streptomycin. All cells were maintained in a 37°C incubator with humidified 5% CO2.

### Lentiviral transduction

Lentiviral particles encoding for hsa-miR-320c-1 (LP-HmiR0470-MR03-0200-S) or control lentiviral particles were purchased from Genecopoeia (Genecopoeia Inc., Rockville, MD, USA). Two hundred thousand HCT116 cells were seeded in complete DMEM in 24-well plate. Twenty-four hours later (~80 confluency), media was removed and then 20*μ*l of crude lentiviral particles in 500*μ*l of DMEM+5% heat-inactivated serum (Invitrogen) and 1% Pen-Strep supplemented with polybrene (8*μ*g/ml; Sigma, St. Louis, MO, USA) was added to the cells. Seventy-two hours later, media was removed and transduced cells were selected with puromycin (1*μ*g/ml, Sigma, St. Louis, MO. USA) for 1 week until stably transduced cells were generated.

### siRNA transfection

The siRNA-negative control, siSOX4, siFOXQ1, siFOXM1, siHMGB3, siRUNX1, siMKI67 and siZWILCH were purchased from Applied Biosystems (Invitrogen, Carlsbad, CA, USA). Transfection was performed using reverse transfection approach as described before [10, 33]. Briefly, 30 nM (final) siRNA was diluted in 50 μl of Opti-MEM (11058-021; Gibco, Carlsbad, CA, USA), whereas 1 μl of Lipofectamine 2000 (Part No: 52758; Invitrogen) were diluted in 50 μl OPTI-MEM. The diluted siRNA, and Lipofectamine 2000 were mixed and incubated at ambient temperature for 20 min. Twenty microliters of transfection mixture was added to the plate and subsequently 5,000 cells in transfection medium (routine culture medium without antibiotics) were added to each well in 60 μl volume. Every experiment was performed in 10 replicates in 96-well cell culture plates with the appropriate controls. The experiment was repeated at least two times. Plates were incubated for the indicated time points, and proliferation or growth inhibition was assessed using the alamarBlue (BUF012B; AbD Serotec, UK) assay.

### Gene expression microarray

RNA isolation, gene and microRNA expression experiments were performed in accordance with our previously published protocols [10, 34]. In brief, RNA was isolated using Total Tissue RNA Purification Kit from Norgen-Biotek Corp. (Thorold, ON, Canada) and were quantified using NanoDrop 2000 (Thermo Scientific, Wilmington, DE, USA). Total RNA was labelled and then hybridized to the Agilent Human SurePrint G3 Human GE 8 × 60 k v16 mRNA microarray chip (Agilent Technologies). All microarray experiments were conducted at the Microarray Core Facility (Stem Cell Unit, Department of Anatomy, King Saud University College of Medicine). Data were subsequently normalized and analyzed using GeneSpring 13.0 software (Agilent Technologies). Pathway analyses were conducted using the Single Experiment Pathway analysis feature in GeneSpring 13.0 (Agilent Technologies). Twofold cut-off with *P* < 0.02 was used. Target prediction was conducted using a built-in feature in GeneSpring 13.0 based on TargetScan database.

### mRNA ad miRNA validation by qRT-PCR

mRNAs expression levels were validated in LV control and miR-320c HCT116 cells using SYBR Green-based qRT-PCR and the Applied Biosystems ViiA 7 Detection system. 500 ng of total RNA was reverse transcribed using High Capacity cDNA Reverse Transcript Kit (Part No: 4368814; ABI) according to the manufacturer's protocol. Relative levels of mRNA were determined from cDNA using real-time PCR (Applied Biosystems ViiA 7 System). Primer sequences used in the current study are listed in Supplementary Table 3. The relative expression level was calculated using −ΔΔCT. β-actin was used as an endogenous control. For miRNA validation, 10 ng of total RNA was reverse transcribed using TaqMan MicroRNA Reverse Transcription Kit (Part No: 4366596, ABI) and relative miRNA expression levels were determined using TaqMan Universal Master Mix II, no UNG (Part No: 4440040, ABI) and hsa-miR-320c primers (ABI). The relative expression level was calculated using −ΔΔCT. RNU44 and RNU48 were used as endogenous control.

### Measurement of cell viability and clonogenic assay

The viability of LV control and miR-320c HCT116 cells was determined using alamarBlue assay as previously described [10]. All assays were carried out with appropriate controls. Briefly, 5000 cells were cultured in a 96-well plate and cell viability was measured at the indicated time points by adding 10% volume alamarBlue assay reagent and measuring absorbance at 570λ. The colony forming ability of HCT116 cells transduced with miR-320c was determined using clonogenic assay as previously described [35, 36]. Briefly, LV control or miR-320c HCT116 cells were seeded in 12-well plates in different serial dilution (1:2 to 1:64). Initial seeding density was 0.015 × 10^6^ cells per well, and incubated at 37°C under 5% CO2 for 10 days. The plates were then washed and stained with Diff-Quik stain set (Siemens), and the plates were scanned and number of colonies was observed under microscope. The fraction of surviving cells was estimated by comparison of miR-320c to LV control cells. The experiment was done twice in duplicate. Furthermore, the clonogenic assay was conducted to examine the effect of 5-Fluorouracil on colony formation in both cells. A total of 1 × 10^6^ cells were seeded in T25 flask. After 48 hours of exposure to 1.5 μM of 5-Fluorouracil, the cells were trypsinized and reseeded in 12-well plates as described above to observe the effect of the drug.

### Immunoblotting

LV control and miR-320c HCT116 cells were lysed using RIPA buffer (Norgen-Biotek Corp.) containing 1 × Halt Protease Inhibitor Cocktail (Pierce Inc., Rockford, IL, USA). Thirty micrograms of total protein were run and blotted using the Bio-Rad V3 Western work flow system according to the manufacturer's recommendation. Immunoblotting was conducted using anti-SOX4 rabbit polyclonal antibody (H-90, dilution 1:1000, Santa Cruz Biotechnology, Santa Cruz, CA), anti-FOXM1 antibody 263C2a (ab58675, dilution 1:400), and anti-FOXQ1 antibody (ab51340, dilution 1:400), both from Abcam (Abcam, Cambridge, MA). Primary antibody was incubated overnight at 4°C. Horseradish peroxidase (HRP)-conjugated goat anti-rabbit (cat. no. 7074, 1:3000 dilution; Cell Signaling) was used as the secondary antibody, whereas HRP-conjugated anti-GAPDH (glyceraldehyde-3-phosphate dehydrogenase) antibody (ab9482, 1:10000; Abcam, Cambridge, MA, USA) was used as the loading control. Quantification of band intensity was conducted using band quantification tool in Image Lab 5.0 software (Bio-Rad, CA, USA).

### Cell migration and proliferation

Real-time measurement of LV control and miR-320c HCT116 cell migration and proliferation was executed using the xCELLigence RTCA DP system (ACEA Biosciences, San Diego, CA). For migration study, cells were starved for 24 hrs in 1% serum media, followed by seeding 0.08×10^6^ cells per well in 16-well microelectronic sensor plate pre-coated with fibronectin (1:500 dilution), two chamber trans-well plates (CIM-plate insert; ACEA Bioscience) containing the respective serum conditions. Medium containing 10% serum (chemo-attractant) and 1% serum (control) was added to the bottom wells.

For proliferation assay, cells were seeded (0.04×10^6^ cells/well) in two chamber plates (E-plate insert; ACEA Bioscience). Proliferation and migration of cells was measured from the interaction of cells with the electrodes on the top chamber and represented as a change in cell index (CI), an arbitrary unit derived from the relative change in electrical impedance across microelectronic sensor arrays. The electrical impedance was captured every 15 min for an experimental duration of ~100 hrs. The rate of migration and proliferation is expressed as the CI or the change in electrical impedance at each time-point. The Cell Index at each time point is defined as (Rn-Rb)/4.6, where Rn is the cell-electrode impedance of the well when it contains cells and Rb is the background impedance of the well with the media alone. Values are expressed as the mean ± SEM of the 3 replica wells from three independent experiments. For conventional migration, the BD transwell migration system with 8 μ pore size was utilized. Inserts were placed in a 24-well plate, and 1.56×10^5^ cells in 1% serum were added to the top of the chamber, and 10% serum added to the bottom chamber. Seventy-two hours later, inserts were fixed and stained with SIEMENNS DIFF-QUICK stain set (Siemens Healthcare Diagnostics), and the number of migrating cells was counted using a light microscope.

### Luciferase reporter assay

For luciferase reporter assays, HEK293 cells were seeded in 12-well plates in 500μl complete DMEM growth medium without antibiotics. Second day when cells reached 80% confluency, cells were transfected with complexes containing control or UTR plasmid (100 ng), pre-miR control or pre-miR-320c (50nM) mixed with lipofectamine 2000 (Part No: 52758; Invitrogen) in Opti-MEM (11058-021; Gibco, Carlsbad, CA, USA). Twenty-four hours after transfection, luciferase activity was measured using the Secrete-Pair™ Dual Luminescence assay kit (Secrete Gaussia luciferase (GLU) and Secreted Alkaline Phosphatase (SEAP); GeneCopeia Inc., USA) according to the manufacturer's instructions while luminescence was subsequently measured using a SpectraMax M5 (Molecular Devices; USA) luminescence reader. The ratio of luminescence intensities of the GLU over SEAP was calculated and normalized to controls.

### Measurement of apoptosis

Fluorescence-based apoptosis was determined in cells after exposure to different concentration of 5-Flurouracil, using acridine orange and ethidium bromide (AO/Etbr) staining method [37]. After treatment, the LV control and miR-320c HCT116 cells were stained with dual fluorescent staining solution containing 100 μg/ml AO and 100 μg/ml EB (AO/EB, Sigma, St. Louis, MO). Cells were washed twice with PBS and were gently mixed with AO/EB (1:100) dye solution for one minute; afterwards, the cells were observed and photographed under a Nikon Eclipse Ti fluorescence microscope. Cells cultured without drug were considered as experiment control. Acridine Orange/Ethidium Bromide staining uses combination of two dyes to visualize cells with aberrant chromatin organization. The differential uptake of AO/EB allows the identification of viable and non-viable cells. Particularly, Acridine Orange was used to visualize the number of cells which has undergone apoptosis.

### *In vivo* tumorigenicity assay in SCID mice

*In vivo* tumor formation was carried out as we previously described [9, 38]. Briefly, Six- to 8-week-old severe combined immunodeficient mice (SCID) were utilized for the xenograft experiments. Ten million LV control or miR-320c HCT116 cells were suspended in PBS and subcutaneously were injected into the right flank of SCID mice. Tumor size was measured twice weekly using a calliper and tumor volumes were calculated as (tumor length × width^2^)/2. At the end of the experiments, primary tumors were excised, fixed in 10% buffered formalin and embedded in paraffin and sectioned. Sections were stained with haematoxylin and eosin.

### TCGA survival data analysis

Kaplan-Meier curve analysis for the expression of SOX4, FOXM1, and FOXQ1 in the TCGA colorectal cancer data set (319 patients) in relation to disease-free survival was conducted as previously described [39, 40]. The log-rank test was used to determine statistical significance for curve comparison.

### Statistical analysis

Statistical analyses and graphing were performed using Microsoft excel 2010 and GraphPad Prism 6.0 software (GraphPad, San Diego, CA, USA). P-values were calculated using the unpaired two-tailed t-test. Pearson's correlation was used to assess the correlation between SOX4, FOXM1, FOXQ1 and different members of the miR-320 family in CRC using the GraphPad Prism software.

## SUPPLEMENTARY MATERIAL FIGURES AND TABLES






