# Supplementary material for: MicroRNA-320 suppresses colorectal cancer by targeting SOX4, FOXM1, and FOXQ1
Source: Oncotarget. 2016 Apr 22;7(24):35789–802. doi: 10.18632/oncotarget.8937 (PMC5094962; doi:10.18632/oncotarget.8937)
Supplement: Supplementary file 1 [file oncotarget-07-35789-s001.pdf]

MicroRNA-320 suppresses colorectal cancer by targeting SOX4, FOXM1, and FOXQ1

Supplementary Material

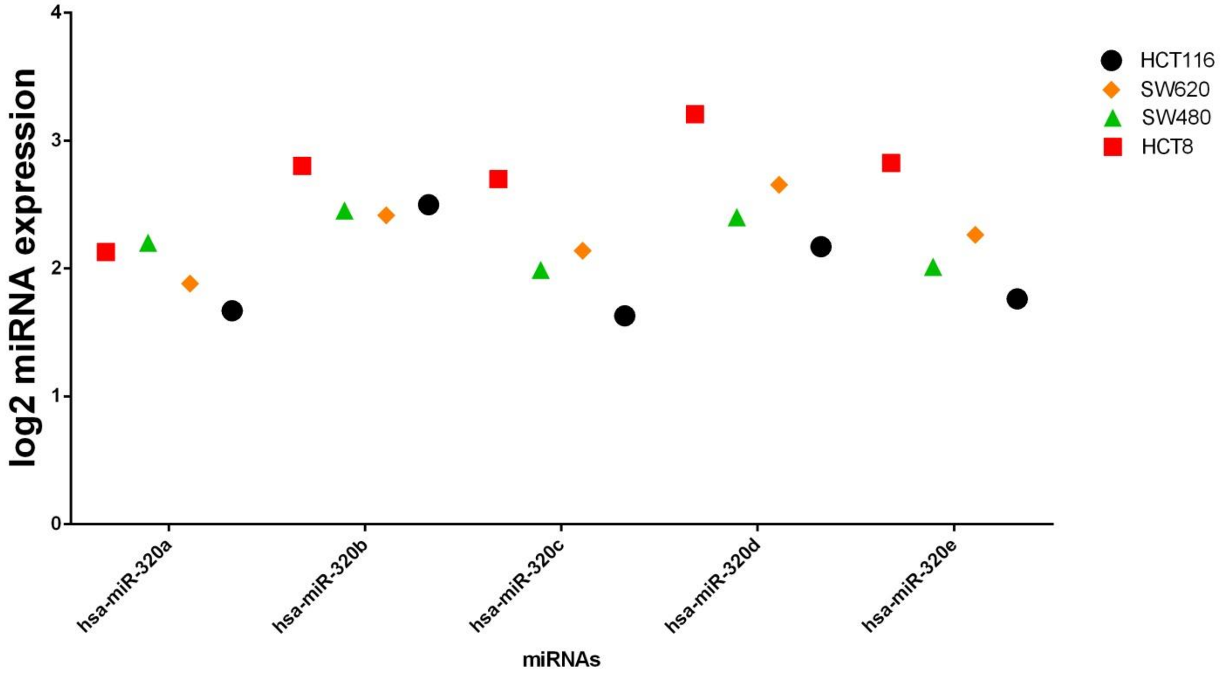

Supplementary figure 1. miR-320 family expression in a panel of CRC cell lines. Data are presented as log2 expression based on microRNA microarray data.

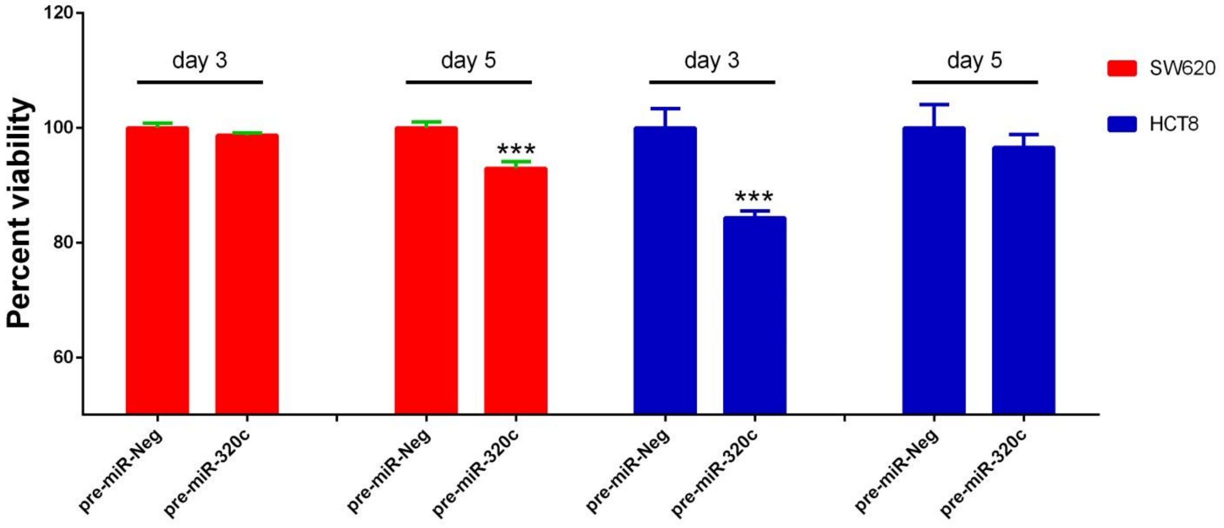

Supplementary figure 2. Exogenous expression of hsa-miR-320c led to significant reduction in cell viability in the SW620 and HCT8 CRC models on days 3 and 5. Data are presented as mean ± S.E., n=8. ***P<0.0005

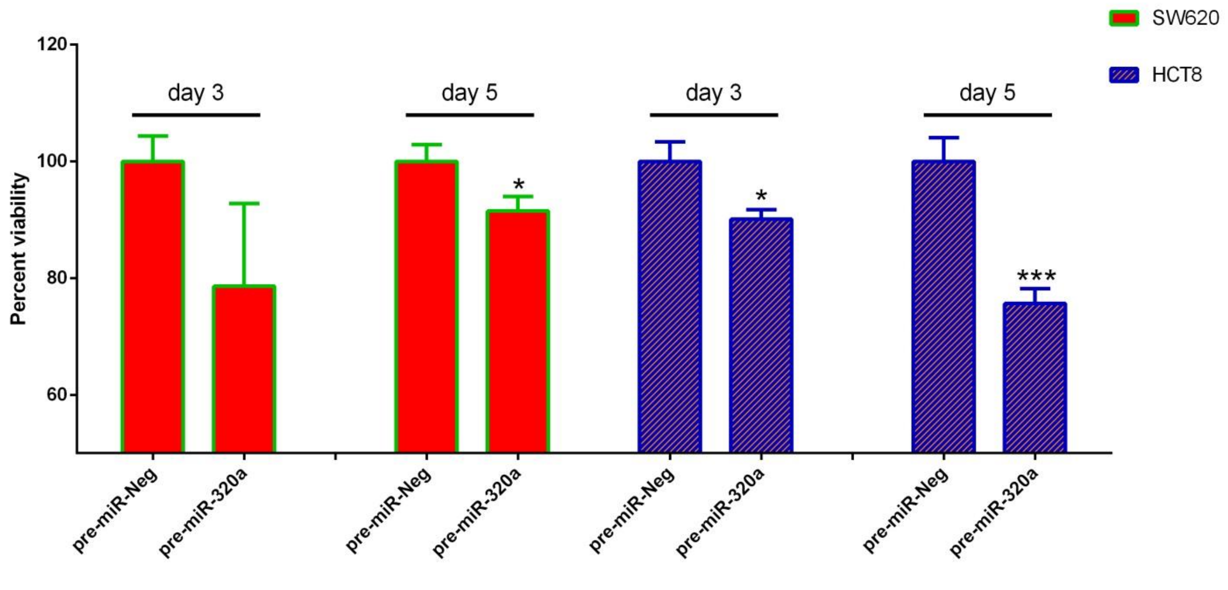

Supplementary Figure 3. Exogenous expression of hsa-miR-320a led to significant reduction in cell viability in the SW620 and HCT8 CRC models on days 3 and 5. Data are presented as mean ± S.E., n=8. *P<0.05; ***P<0.0005.

Supplementary Table 3. List of SYBR green primers used in current study

No	Name	Sequence
1	SOX4	F 5′ GTTTCGCTGTCGGGTCTCTA
		R 5′ GAAGGGAGCTGGTAATGGCA
2	FOXQ1	F 5′ CGGAAGAGGACTCCGGAAAAG
		R 5′ GTCGTACCCTCTTCCTTCGC
3	FOXM1	F 5` TTTATCAGTGCTGCTAGCTGAGG
		R 5` CCCCAGGCTGGATTTCTTCC
4	HMGB3	F 5` ACTAGCGAACAATACAGTCAGGA
		R 5` GCATAAGCGGACATCTTGCC
5	RUNX1	F 5` CTTCACAAACCCACCGCAAG
		R 5` CTGCCGATGTCTTCGAGGTT
6	MKI67	F 5` ATACGTGAACAGGAGCCAGC
		R 5` AGGCCTTGGAATCTTGAGCTTT
7	ZWILCH	F 5` GTACCGCTCTCACATTGGGG
		R 5` GCACTTGGACATCAGCCTCATA
8	E2F1	F 5` CATCAGTACCTGGCCGAGAG
		R 5` CCCGGGGATTTCACACCTTT
9	B-actin	F 5` TCAAGATCATTGCTCCTCCTGAG
		R 5` ACATCTGCTGGAAGGTGGACA
